# FGF10/FGFR2 signal induces cell migration and invasion in pancreatic cancer

**DOI:** 10.1038/sj.bjc.6604473

**Published:** 2008-07-01

**Authors:** S Nomura, H Yoshitomi, S Takano, T Shida, S Kobayashi, M Ohtsuka, F Kimura, H Shimizu, H Yoshidome, A Kato, M Miyazaki

**Affiliations:** 1 The Department of General Surgery, Graduate School of Medicine, Chiba University, 1-8-1 Inohana, Chuo-ku, Chiba city, Chiba 260-8670, Japan

**Keywords:** pancreatic cancer, fibroblast growth factor 10, fibroblast growth factor receptor 2, cancer stromal cell

## Abstract

Pancreatic cancer has one of the highest mortalities among all malignancies and there is an urgent need for new therapy. This might be achieved by resolving the detailed biological mechanism, and in this study we examined how pancreatic cancer cells develop aggressive properties by focusing on signalling through the fibroblast growth factor (FGF)10 and FGF receptor (FGFR)2, which play important roles in pancreatic organogenesis. Immunostaining of pancreatic cancer tissues showed that FGFR2 was expressed in cancer cells, whereas FGF10 was expressed in stromal cells surrounding the cancer cells. Patients with high FGFR2 expression in cancer cells had a shorter survival time compared to those with low FGFR2 expression. Fibroblast growth factor 10 induced cell migration and invasion of CFPAC-1 and AsPC-1 pancreatic cancer cells through interaction with FGFR2-IIIb, a specific isoform of FGFR2. Fibroblast growth factor 10 also induced expression of mRNA for membrane type 1-matrix metalloproteinase (MT1-MMP) and transforming growth factor (TGF)-*β*1, and increased secretion of TGF-*β*1 protein from these cell lines. These data indicate that stromal FGF10 induces migration and invasion in pancreatic cancer cells through interaction with FGFR2, resulting in a poor prognosis. This suggests that FGF10/FGFR2 signalling is a promising target for new molecular therapy against pancreatic cancer.

Pancreatic cancer has one of the highest mortalities among all malignancies, and is the fourth most common cause of cancer death in the United States and the fifth in Japan (Li *et al*, 2004; Willett *et al*, 2005). Although significant advances are now being made into the management of the disease, the 5-year survival rate still remains poor (Willett *et al*, 2005; Ghaneh *et al*, 2007). Therefore, there is an urgent need for new therapies for pancreatic cancer, based on an improved understanding of the molecular biology of the disease. The high mortality rate of pancreatic cancer is, in part, owing to difficulties of early diagnosis, the high incidence of metastatic disease at the time of diagnosis, and rapid progression of the disease. In addition, although newer adjuvant modalities are greatly increasing the prognosis (Ghaneh *et al*, 2008), most patients who undergo the surgery eventually relapse and die of the disease, even with curative resection (Li *et al*, 2004; Willett *et al*, 2005).

An understanding of the mechanisms underlying the biological aggressiveness of pancreatic cancer may be key for development of new therapies. Therefore, in this study we examined the molecular mechanisms underlying cellular invasion and metastasis of pancreatic cancer cells. Cellular and genetic studies have shown that tumour growth is not determined by malignant cancer cells alone, but also by cells in the tumour stroma. Supply of oxygen and nutrients by endothelial cells of blood vessels are critical for maintenance of the tumour microenvironment, and stromal fibroblasts are the principal source of extracellular matrix, which serves as a scaffold for cancer cells (Kalluri and Zeisberg, 2006). In addition, recent studies have revealed more active roles of stromal cells in tumour initiation and progression through direct interaction with tumour cells. For example, stromal cell-derived factor-1 (SDF-1/CXCL12) released from fibroblasts promotes cancer cell proliferation through a specific receptor, CXCR4, in several types of malignancies, including breast cancer (Orimo *et al*, 2005) and pancreatic cancer (Koshiba *et al*, 2000; Marchesi *et al*, 2004). Immune cells also play important roles in cancer progression (Pollard, 2004); for example, tumour-associated macrophages induced by colony-stimulating factor 1 promote invasiveness of cancer cells (Lin *et al*, 2001). Given this background, we hypothesized that stromal cell–cancer cell interactions have an important role in acquisition of the aggressive character by pancreatic cancer, and we examined signalling molecules that may be associated with this mechanism.

The molecular mechanisms underlying carcinogenesis are often similar to those in organogenesis. Interactions between stromal and parenchymal cells are important during organ development, and signals from stromal cells regulate epithelial cell growth and differentiation in pancreatic development. The classical tissue recombinant study by Golosow and Grostein (1962) showed that growth and morphogenesis of the developing pancreas depend on mesenchymal interactions, and more recently advances in molecular biology have allowed the molecular basis of this interaction to be established. We have shown that signals from endothelial cells and mesenchymal cells surrounding the pancreatic bud are crucial for initiation of pancreatic development from endoderm (Yoshitomi and Zaret, 2003; Jacquemin *et al*, 2006). Especially, we found that fibroblast growth factor-10 (FGF10) from mesenchymal cells maintained expression of Ptf1a, a critical transcription factor for initiation of pancreatic development, in pancreatic progenitor cells (Jacquemin *et al*, 2006). Mice deficient in FGF10 or the FGF receptor-2 (FGFR2)/IIIb isoform, the specific receptor for FGF10 (Igarashi *et al*, 1998), show impaired pancreatic development (Bhushan *et al*, 2001; Pulkkinen *et al*, 2003). However, it is unknown if FGF10/FGFR2-IIIb-signalling is associated with carcinogenesis in pancreatic cancer. In this study, we show that FGF10/FGFR2-signalling has an important role in pancreatic cancer progression, and we suggest that these results may lead to a new therapy and a better prognosis for patients with pancreatic cancer.

## Materials and methods

### Patients and tissue samples

Pancreatic cancer tissues were obtained from 76 pancreatic cancer patients who underwent curative surgical resection in the Department of General Surgery, Chiba University Hospital, Chiba, Japan, from June 2001 to April 2006. All patients were diagnosed histologically as primary invasive pancreatic ductal carcinoma. The patient characteristics are summarised in Table 1. The study protocol was approved by the Ethics Committee of our institute and written informed consent was obtained from all patients.

### Immunohistochemistry

Paraffin-embedded tissues were cut into 4 *μ*m serial sections and deparaffinised. The sections were placed in citrate buffer (10 mmol l^−1^ pH 6.0) with 0.2% Tween 20 and boiled in a microwave oven (two times × 6 min) to retrieve the antigen. They were then rinsed and blocked in 10% H_2_O_2_ solution with methanol for 10 min. Next, the sections were incubated with goat anti-human FGF10 polyclonal antibody (R&D Systems, Minneapolis, MN, USA) at 1 : 20 dilution, mouse anti-human FGFR2 monoclonal antibody (R&D Systems) at 1 : 10 dilution, or rabbit anti-human CD3 monoclonal antibody (ready-to-use without dilution) (Thermo Fisher Scientific Anatomical Pathology, Fremont, CA, USA) overnight at 4°C. They were then rinsed in PBS and incubated for 60 min with a secondary antibody labelled with streptoavidin–biotin–peroxidase for goat polyclonal antibody (DAKO LSAB+™ System, DAKO, Glostrup, Denmark), or dextran polymer-peroxidase for mouse monoclonal antibody (DAKO EnVision™ System, DAKO). For detection of anti-CD3 antibody, CSA II biotin-free catalysed amplification system with rabbit link (DAKO) was used. The bound complex was visualised using diaminobenzidine liquid chromogen (Dako) and counterstained with haematoxylin. Goat polyclonal IgG (Santa Cruz Biotechnology. Santa Cruz, CA, USA) at an optimal dilution was used as a negative control.

### Cell lines and culture conditions

AsPC-1 cells were maintained in RPMI-1640 medium (Invitrogen, Carlsbad, CA, USA) and MIA PaCa-2, PANC-1 and CFPAC-1 cells were cultured with DMEM medium (Sigma-Aldrich, St Louis, MO, USA). All cell lines were incubated in a humidified atmosphere containing 5% CO_2_ at 37°C. Each medium contained 10% fetal bovine serum (Invitrogen), 100 U ml^−1^ penicillin and 0.1 mg ml^−1^ streptomycin sulphate (Sigma-Aldrich).

For some experiments, cells were seeded (1 × 10^6^ cells in 2 ml medium per well) in 6-well plates, and cultured for 24 h. Then cells were cultured with serum-free medium for another 24 h and changed to new serum-free medium with recombinant human FGF10 (R&D systems) or/and FGFR2-IIIb/IgG Fc Chimera (R&D systems) for indicated time in each experiments. Cells and medium were harvested for further experiments.

Transforming growth factor *β* 1 (TGF-*β*1) concentration was measured using Quantikine Human TGF-*β*1 immunoassay kit (R&D systems).

### Reverse transcription–PCR

Total RNA was extracted from cultured cells, pancreatic cancer tissues and adjacent normal tissues with an RNeasy Mini Kit (QIAGEN GmbH, Hilden, Germany), according to the manufacturer’s instructions. cDNA was synthesised from 1 *μ*g of total RNA with SuperScript™ III First-Strand Synthesis SuperMix for reverse transcription (RT)–PCR (Invitrogen). Polymerase chain reaction was performed with the following primer sets: FGF10, forward 5-ACATTGTGCCTCAGCCTTTC-3, reverse 5-CCCCTTCTTGTTCATGGCTA-3; FGFR2-IIIb, forward 5-TATATAGGGCAGGCAACCA-3, reverse 5-GCTGAAGTCTGGCTTCTTGG-3; FGFR2-IIIc, forward 5-GTGCTTGGCGGGTAATTCTA-3, reverse 5-GCTGAAGTCTGGCTTCTTGG-3; and glyceraldehyde-3-phosphate dehydrogenase (GAPDH), forward 5-GTCAGCCGCATCTTCTTTTG-3, reverse 5-TTCACACCCATGACGAACAT-3. The RT–PCR conditions for FGF10, FGFR2-IIIb, FGFR2-IIIc and GAPDH were as follows: 94°C for 2 min, 40 cycles at 94°C for 15 s, 58°C for 30 s, and 72°C for 30 s, with an extension step of 7 min at 72°C at the end of the last cycle.

### Quantitative RT–PCR

Quantitative RT–PCR was performed as described previously (Mitsuhashi *et al*, 2003). Primers for 18 genes related to cancer invasion and motility have been described by Ide *et al* (2006). The mRNA levels of these genes (E-Cadherin, N-Cadherin, Snail, MMP-1, MMP-2, MMP-7, MMP-9, MT1-MMP, TIMP-2, uPA, TGF-*β*1, HGF, c-Met, RhoA, CD44, Integrin-*α*4, Integrin-*β*4, and VEGF-A) were determined as the absolute number of copies normalised against the GAPDH mRNA copy number (Mitsuhashi *et al*, 2003). These experiments were performed three times independently.

### Cell migration assay

A migration assay was performed in 12-well plates using a Quantitative Cell Migration™ Assay Kit (Chemicon International, Temecula, CA, USA) with an 8.0 *μ*m pore size collagen-coated chamber membrane. The cells were seeded (1 × 10^5^ cells in 0.3 ml of serum-free medium) in the upper chamber and cultured for 24 h for attachment. The medium was then replaced by fresh serum-free medium for another 24 h, before addition of recombinant human FGF10 (100 ng ml^−1^) (R&D Systems) to the lower chamber. In some experiments, recombinant human FGFR2-IIIb/IgG Fc Chimera (500 ng ml^−1^) (R&D Systems) was also added to the lower chamber. The cells were incubated for 12 h and the number of cells that passed through the membrane was counted according to the manufacturer’s instructions. All experiments were performed in triplicate and independently at least three times.

### Cell invasion assay

An invasion assay was performed in 24-well plates using a BD Biocoat™ Matrigel™ Invasion Chamber (BD Biosciences, Bedford, MA, USA) with an 8.0 *μ*m. pore size polyethylene terephthalate (PET) membrane coated with Matrigel. The inserts were rehydrated by adding 0.5 ml of warm culture medium at 37°C for 2 h. The cells were seeded (5 × 10^5^ cells in 0.5 ml of serum-free medium) in the upper chamber and cultured as described in the method for the migration assay. The number of seeded cells, culture conditions and other items were also similar to those for the migration assay. The cells were incubated for 24 h and the number of cells that passed through the membrane was counted according to the manufacturer’s instructions. All experiments were performed in triplicate and independently at least three times.

### Statistical analysis

Values are expressed as means±s.d. The distribution of categorical data for FGFR2 immunostaining in tissue samples and for clinicopathological characteristics were assessed by a *χ*^2^ test and a Fisher's exact test. Survival time was calculated using the Kaplan–Meier method and compared by log-rank test. Cell migration and cell invasion data were analysed using a Student’s *t*-test and a Mann–Whitney *U*-test. Statistical significance was assumed for *P*<0.05.

## Results

### Expression of FGFR2 in pancreatic cancer cells and FGF10 in cancer stromal cells

**Figure 1:**
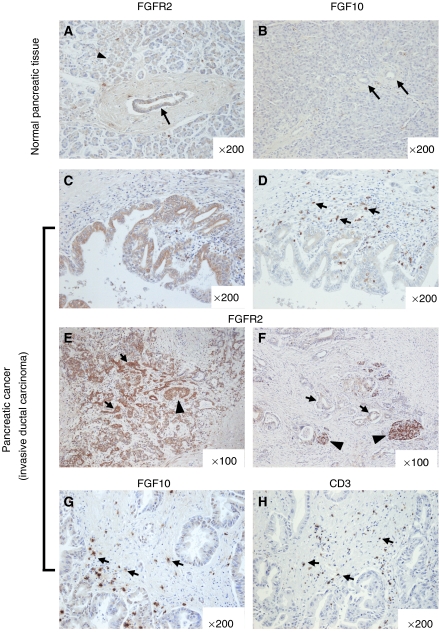
Expression patterns of FGFR2 and FGF10 in normal pancreas and pancreatic cancer. The magnification is shown in the right bottom corner of each figure. (**A** and **B**) Immunostaining of FGFR2 (**A**) and FGF10 (**B**) in normal pancreas, showing that FGFR2 is expressed weakly in ductal cells (**A**, arrow) and acinar cells (**A**, arrow head), and that no obvious FGF10 staining is found in normal pancreatic tissue, including ductal cells (**B**, arrows). (**C** and **D**) Immunostaining of FGFR2 (**C**) and FGF10 (**D**) in pancreatic cancer tissues, showing that FGFR2 is expressed in cancer cells (**C**), whereas FGF10 is expressed in scattered cells in the stroma surrounding cancer cells (**D**, arrows). (**E** and **F**) Immunostaining of FGFR2 in pancreatic cancer cells. (**E**) Representative result from the FGFR2 high expression group, indicating higher FGFR2 expression in cancer cells (arrows) compared with islets (arrow head). (**F**) Representative result from the FGFR2 low expression group, showing lower FGFR2 expression in cancer cells (arrows) compared with islet (arrow heads). (**G** and **H**) Immunostaining of FGF10 (**G**) and CD3 (**H**), marker for T cell. Fibroblast growth factor 10 and CD3 are both expressed in scattered cells with similar cell shape in the stroma surrounding cancer cells (arrows).

To examine the expression pattern of FGFR2 and FGF10 in pancreatic cancer tissues, we performed immunohistochemical staining of 76 tissue samples of invasive pancreatic ductal carcinoma and of normal pancreatic tissues. FGF receptor-2 immunoreactivity was weak to moderate in pancreatic ductal cells in normal tissues (Figure 1A; arrow) and acinar cells (Figure 1A; arrowhead), as well as in islet cells (data not shown), as described previously (Ishiwata *et al*, 2002). On the other hand, immunostaining of FGF10 did not occur in normal pancreatic tissue, as also described previously (Ishiwata *et al*, 2002) (Figure 1B).

In pancreatic cancer tissues, cancer cells expressed FGFR2 at various levels (Figure 1C), but did not express FGF10 (Figure 1D). Pancreatic cancer tissue often contains a few islet cells (Figure 1E and 1F; arrowheads) and we compared the expression levels of FGFR2 in cancer cells and islets. In 39 cases (51.3%), FGFR2 immunoreactivity in cancer cells was stronger than in islets (high expression group, Figure 1E). In the other 37 cases (48.7%), FGFR2 immunoreactivity was not found or was faint in cancer cells, and was weaker than in normal islet cells (low expression group, Figure 1F). There was no correlation between FGFR2 immunoreactivity levels and tumour histological findings (Table 1).

Pancreatic cancer cells did not express FGF10 in any samples (Figure 1D), but scattered cells in stroma surrounding the cancer cells showed strong expression of FGF10 (Figure 1D; arrows). Fibroblast growth factor 10-positive stromal cells in cancer tissues were found in 42 cases (55.3%), and interestingly, were mainly localised close to cancer cells (Figure 1D and 1E). Moreover, of the 42 cancer tissue samples with FGF10-positive stromal cells, 29 (69.0%) showed high FGFR2 expression in cancer cells, and there was a significant correlation between the presence of FGF10-positive stromal cells and high FGFR2 expression in cancer cells (*P*=0.013).

Next we examined which kind of cancer stromal cells expressed FGF10. We stained sequential sections with antibody against FGF10 and stromal cell markers (CD68; macrophage marker, *α*-smooth muscle actin; activated fibroblast marker, CD3; T-cell marker). Within them, CD3-positive stromal cells, T-cells, were located similar to FGF10-positive stromal cells and also has similar cell shapes (Figure 1G and 1H; arrows). However, due to the technical difficulties, we could not demonstrate that FGF10-expressing cells were identical with T-cells.

Overall, the results show that FGFR2 is expressed in pancreatic cancer tissue, and that its ligand, FGF10, is expressed in stromal cells.

### FGFR2 expression levels correlates with prognosis of pancreatic cancer patients

**Figure 2:**
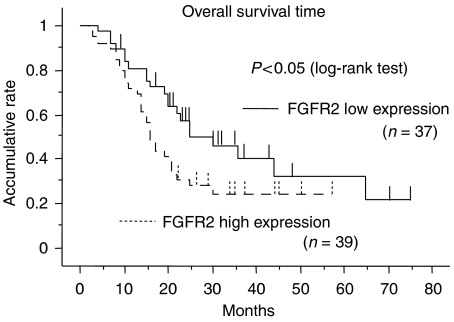
Kaplan–Meier survival curves for patients with high and low FGFR2 expression. Patients with low FGFR2 expression had a significantly longer overall survival time compared to those with high FGFR2 expression (*P*<0.05 by log-rank test).

Next, we examined whether strong expression of FGFR2 in cancer cells correlated with patient prognosis. The 76 patients were divided into two groups according to the expression level of FGFR2 in cancer cells (the high and low expression groups in Table 1). The expression level of FGFR2 was not correlated with clinicopathological factors such as sex, age and pathological stage (Table 1). There were no statistical differences in the resection status of both groups. Also, the ratios of patients who received adjuvant chemotherapy (using gemcitabine) were similar in both groups, indicating that patients in both groups were treated with similar therapeutic approaches after surgery (Table 1). Interestingly, Kaplan–Meier analysis showed that patients with high FGFR2 expression had a significantly shorter overall survival time compared to those with low expression levels (Figure 2) (*P*=0.047 by log-rank test). Moreover, patients with high FGFR2 expression had significantly more nodal invasion, a larger tumour size, and a worse UICC Stage (Table 2; *P*=0.0263, *P*=0.0469, and *P*=0.022, respectively, by *χ*^2^-test). There was no significant correlation between survival time and the presence of FGF10 in stromal cells in cancer tissue (data not shown).

### FGF10/FGFR2-IIIb signalling induces cell migration and invasion

The expression pattern of FGF10 and FGFR2 in cancerous tissue and the poor prognosis of patients with high FGFR2 expression in cancer cells indicate that a stromal cell–epithelial cell interaction through FGF10/FGFR2 signalling might induce the malignant properties of pancreatic cancer. To examine this hypothesis, we analysed the effects of FGF10 on the proliferation, invasion and migration of pancreatic cancer cells. For this purpose, four pancreatic cancer cell lines were used: MIA PaCa-2, PANC-1, CFPAC-1 and AsPC-1 cells.

**Figure 3:**
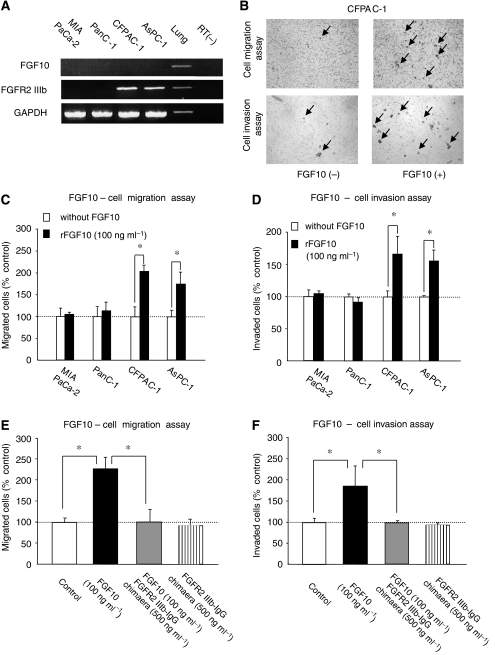
Fibroblast growth factor 10 induces cell migration and invasion in pancreatic cell lines with FGFR2-IIIb expression. (**A**) RT–PCR analysis of FGF10 and FGFR2-IIIb in four pancreatic cell lines and cDNA obtained from normal lung tissue as a positive control. None of the cell lines express FGF10. MIA PaCa-2 and PanC-1 cells do not express FGFR2-IIIb, but CFPAC-1 and AsPC-1 do express this gene. (**B**) Representative results of cell migration (upper panels) and invasion (lower panels) for CFPAC-1 cells. Representative migrated and invaded cells are indicated with arrows. (**C** and **D**) Cell migration (**C**) and invasion (**D**) assay of all four cell lines cultured without (white column) or with (black column) FGF10 (100 ng ml^−1^). FGF10-induced cell migration and invasion in CFPAC-1 and AsPC-1 cells, but not in MIAPaCa-2 and PanC-1 cells. The numbers of migrated or invaded cells cultured with FGF10 are shown relative to a value of 100% for cell migration without ligand. (**E** and **F**) Inhibition of FGFR2-IIIb signalling by an FGFR2-IIIb/IgG chimera in CFPAC-1 cells. Migration (**E**) and invasion (**F**) assay. The numbers of migrated or invaded cells are shown relative to a value of 100% for cells cultured without FGF10 or chimera (control; white column). FGF10-induced migration and invasion in CFPAC-1 cells (black column). Addition of the FGFR2-IIIb/IgG chimera completely eliminated the effects of FGF10 (grey column), whereas the chimera itself did not affect cell migration and invasion of CFPAC-1 cells (striped column). ^*^P<0.05.

First, we examined whether these cell lines expressed FGFR2 and FGF10. Reverse transcriptase–PCR analysis showed that all four cell lines did not express FGF10 mRNA, consistent with the results of immunostaining showing FGF10 expression in stromal cells, but not in cancer cells, in pancreatic cancer tissue (Figure 3A). Fibroblast growth factor 10 activity is dependent on its binding to the FGFR2-specific isoform, FGFR2-IIIb (Igarashi *et al*, 1998). Therefore, a primer set for FGFR2-IIIb was designed with a 5 primer for its specific exon in the FGFR2 gene. Reverse transcriptase–PCR analysis with this primer showed that CFPAC-1 and AsPC-1 cells expressed the FGFR2-IIIb isoform, whereas the other two cell lines did not do so (Figure 3A).

To examine cancer cell proliferation, the cells were stimulated with various concentrations of FGF10 (10–200 ng ml^−1^), but no effect of FGF10 was observed in any of the four cell lines (data not shown). Next, we examined whether FGF10 affects migration or invasion of pancreatic cancer cells. Representative results from the cell migration and invasion assays are shown in Figure 3B. Interestingly, FGF10 stimulated cell migration and invasion of cells that expressed FGFR2-IIIb (CFPAC-1 and AsPC-1), but not of cells without expression of the specific receptor (MIA PaCa-2 and PANC-1) (Figure 3C and 3D). For CFPAC-1 cells, migration was almost doubled (Figure 3C) and invasion was increased by 1.5 times (Figure 3D) following stimulation with FGF10 (100 ng ml^−1^). Similar results were obtained for AsPC-1 cells (Figure 3C and 3D).

To confirm that the effects of FGF10 on pancreatic cells were mediated through FGFR2-IIIb, we used a molecular hybrid including the FGFR2-IIIb extracellular domain and the carboxy-terminal Fc region of human IgG (recombinant human FGFR2-IIIb/IgG Fc Chimera). Such hybrids inhibit signalling through the FGFR2-IIIb receptor by antagonising ligand binding (Jung *et al*, 1999). Stimulated migration of CFPAC-1 cells by 100 ng ml^−1^ FGF10 was completely inhibited by addition of FGFR2-IIIb/IgG Fc Chimera (500 ng ml^−1^) (Figure 3E; compare the second and third columns). The effects of the chimera were due to inhibition of signalling through FGFR2-IIIb, rather than to a direct effect of the chimera on the cells, as addition of chimera alone did not affect cell migration (Figure 3E; compare the first and fourth columns). Invasion of CFPAC-1 cells stimulated by FGF10 was also inhibited by FGFR2-IIIb/IgG Fc Chimera (Figure 3F). Overall, these results indicate that FGF10 stimulates migration and invasion of cancer cells through its specific receptor, FGFR2-IIIb.

### Upregulation of MT1-MMP and TGF-*β*1 mRNA by FGF10

**Figure 4:**
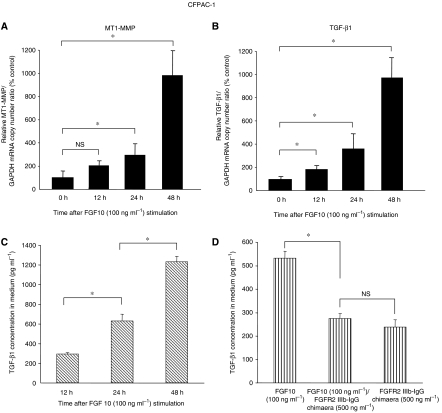
Fibroblast growth factor 10 induces expression of mRNA for MT1-MMP and TGF-*β*1 in CFPAC-1 cells. The figure shows the relative copy numbers of MT1-MMP (**A**) and TGF-*β*1 (**B**) mRNA in CFPAC-1 cells cultured with 100 ng ml^−1^ of FGF10 for the indicated times. The MT1-MMP and TGF*β*1 mRNA/GAPDH mRNA copy number ratios are shown relative to those of cells without FGF10 stimulation (0 h). The concentration of TGF-*β*1 protein in medium also increased in time-dependent manner in CFPAC-1 cells 48 h after addition of FGF10 (100 ng ml^−1^) (**C**). FGF receptor-2-IIIb/IgG chimera (500 ng ml^−1^) inhibited this TGF-*β*1 secretion by FGF10, whereas chimera alone did not affect TGF-*β*1 secretion by itself (**D**). ^*^P<0.05; NS=not significant.

To examine the molecular mechanisms through which FGF10 signalling induces migration and invasion of pancreatic cancer cells with FGFR2-IIIb expression, we analysed whether FGF10 stimulation induced mRNA expression of 18 genes related to cell mobility in CFPAC-1 cells (see Materials and methods for the 18 genes). CFPAC-1 cells were cultured with recombinant human FGF10 (100 ng ml^−1^) for 12, 24 and 48 h, and then total RNA were extracted and the mRNA levels of the 18 genes were analysed by quantitative RT–PCR. Among these genes, FGF10 upregulated the expression levels of MT1-MMP (Figure 4A) and TGF-*β*1 (Figure 4B) mRNAs in a time-dependent manner. In CFPAC-1 cells, the mRNA expression levels for both genes were almost 10 times higher after FGF10 stimulation for 48 h compared with the level before stimulation. The concentration of TGF-*β*1 in culture medium was also upregulated in time-dependent manner in CFPAC-1 cells (Figure 4C) and also in AsPC-1 cells (data not shown). The secretion of TGF-*β*1 by FGF10 stimulation in CFPAC-1 cells was inhibited by FGFR2-IIIb/IgG chimera (Figure 4D), indicating FGF10-induced TGF-*β*1 secretion through FGFR2.

## Discussion

In this study, we investigated the molecular mechanisms underlying the aggressiveness of pancreatic cancer, and found that FGF10/FGFR2-IIIb-signalling plays an important role in inducing migration and invasion of pancreatic cancer cells. FGF receptor-2-IIIb is a splice variant of FGFR2 that is predominantly expressed by cells of epithelial origin and is involved in proliferation of these cells, whereas other FGFR2 variant transcripts are detected in mesenchymal cells (Miki *et al*, 1992). Ishiwata *et al* (1998) first showed that the FGFR2-IIIb isoform of the FGF receptor is expressed in pancreatic cancer cells. We confirmed this result by immunostaining of 76 resected pancreatic cancer tissues, and moreover, showed that the expression level of FGFR2-IIIb is correlated with prognosis and the incidence of nodal involvement. These findings indicate that signalling through FGFR2-IIIb may induce malignant potential, and especially, may increase the metastatic ability of pancreatic cancer cells.

FGFR2-IIIb was first identified as a high-affinity receptor of FGF7 (keratinocyte growth factor) (Miki *et al*, 1992), and it can also be activated by FGF10, which has strong sequence homology with FGF7 (Igarashi *et al*, 1998). Our results show that FGF10 is expressed in stromal cells scattered around pancreatic cancer cells, suggesting a possible interaction with cancer cells expressing FGFR2-IIIb. To our knowledge, this is the first report of the expression pattern of FGF10 in pancreatic cancer. We also show that FGF10-positive cells and CD3-positive T cells exist in a similar location and have a similar shape. According to these data, we hypothesize that FGF10 is expressed in T cells surrounding pancreatic cancer cells. Supporting this, during wound healing, T cells bearing *γδ* T cell receptors are an important source of FGF7 and FGF10, which activate epithelial cell proliferation (Jameson *et al*, 2002). Further studies are required for confirming this hypothesis.

Despite the high homology, the function of FGF10 differs slightly from that of FGF7. We found that FGF10 induced cell migration and invasion in pancreatic cancer cells, but had no effect on cell proliferation. Alderson *et al* (2002) also found that FGF10 does not affect cell proliferation in several types of cancer cells, whereas Niu *et al* (2007) recently showed that FGF7 stimulates cell proliferation, in addition to cell migration and invasion, in pancreatic ductal epithelial cells. These two genes also have different expression patterns in pancreatic cancer. Cho *et al* (2007) showed that FGF7 is expressed in pancreatic cancer cells themselves and acts in an autocrine manner, whereas our results showed FGF10 expression in stromal cells of pancreatic cancer, indicating a paracrine FGF10/FGFR2-IIIb interaction. Further studies are required to understand how these ligands, which share the same receptor on cancer cells, are orchestrated to induce malignant properties in pancreatic cancer.

To understand how FGF10/FGFR2-IIIb signalling induces cell migration and invasion of pancreatic cancer cells, we examined whether FGF10 influences the expression of genes related to cell mobility. Interestingly, FGF10 induced expression of membrane type 1-matrix metalloproteinase (MT1-MMP) and transforming growth factor (TGF)-*β*1 mRNA in CFPAC-1 cells, and these genes may lead, at least in part, to cell migration and invasion of cancer cells. The metalloproteinases are known to involve cell invasion ability. Within several types of metalloproteinases and their inhibitor that we examined (MMP-1, 2, 7, 9, MT1-MMP, TIMP-2), only the mRNA expression of MT1-MMP was upregulated by FGF10 in CFPAC-1 cells. Membrane type 1-matrix metalloproteinase was originally found as a metalloproteinase with a transmembrane domain in homology screening for the MMP conserved domain (Sato *et al*, 1994). The MT1-MMP protein induces invasive activity by degrading extracellular matrix surrounding epithelial cells through its proteinase activity, or by activating other proteinases (Seiki, 2003). In pancreatic cancer, enhanced MT1-MMP expression is particularly observed in metastatic lesions (Maatta *et al*, 2000). These facts may indicate that induction of MT1-MMP is one of the mechanisms through which FGF10 induces cell invasion activity. We are trying to examine if the proteinase activity of MT1-MMP is increased by FGF10 stimulation in pancreatic cancer cells.

TGF-*β*1 is also an important regulator of cell invasion and migration activity, and is frequently overexpressed in pancreatic cancer, with the expression level associated with an advanced tumour stage and a poor prognosis (Friess *et al*, 1993a, 1993b). Moreover, TGF-*β*1 is an important regulator of the epithelial mesenchymal transition, in which epithelial cells disassemble their junctional structures, start expressing mesenchymal cell proteins, remodel the extracellular matrix, and become migratory (Moustakas and Heldin, 2007). As a result, cancer cells acquire metastatic properties (Oft *et al*, 1998; McEarchern *et al*, 2001), suggesting that FGF10/FGFR2-IIIb-signalling may promote migration of pancreatic cancer cells through induction of TGF-*β*1 expression. We also found that FGF10 induced not only mRNA expression of TGF-b1 but also the secretion of this protein through signalling through FGFR2-IIIb in CFPAC-1 cells. This strongly supports the hypothesis and it should be confirmed in future.

In conclusion, our results indicate an important role for the interaction between stromal cells and parenchymal cells mediated by FGF10/FGFR2-IIIb signalling in pancreatic cancer. This suggests that FGF10 and FGFR2-IIIb are promising candidates as target molecules for new therapy against pancreatic cancer, and that therapeutic agents directed against these molecules may improve the prognosis of patients with this disease.

## Figures and Tables

**Table 1:** Characteristics of pancreatic cancer patients in IHC analysis

		**FGFR2**			**FGF10**		
	**Total**	**Low**	**High**	***P*-value**	**Low**	**High**	***P*-value**
	76	37	39		34	42	
*Sex*				NS			NS
M	44	18	26		19	25	
F	32	19	13		15	17	
							
*Age (years)*				NS			NS
Mean	65.0	65.0	64.5		65.0	64.5	
±s.d.	±9.2	±9.3	±9.4		±9.3	±8.8	
							
Stage				NS			NS
IA	2	2	0		2	0	
IB	2	2	0		2	0	
IIA	15	9	6		4	11	
IIB	53	22	31		24	29	
III	4	2	2		2	2	
							
*Histology*				NS			NS
*Tubular adenocarcinoma*							
Well.	7	3	4		4	3	
Mod.	47	26	21		19	28	
Poor.	13	5	8		9	4	
*Invasive carcinoma derived from intraductal tumour*	4	2	2		0	4	
Anaplastic carcinoma	3	1	2		2	1	
Adenosquamous carcinoma	2	0	2		0	2	
							
*Resection status*							
Negative	51	26	25	NS	23	29	NS
Positive	25	11	14		12	13	
							
*Adjuvant chemotherapy*							
−	21	9	12	NS	9	12	NS
+	55	28	27		25	30	

Mod=moderately differentiated; NS=no significant; Poor=poorly differentiated; s.d.=standard deviation; Well=well differentiated.

Patient stage was determined according to UICC TNM classification.

**Table 2:** Clinico-pathological features of pancreatic cancer patients in FGFR2-IHC analysis

		**FGFR2 expression of IHC**		
	**Total**	**Low expression**	**High expression**	
	**76**	**37**	**39**	***P*-value**
T (1,2,3/ 4)	42/34	23/14	19/20	NS
N (0/ 1,2,3)	20/56	14/23	6/33	0.0263
M (0/ 1)	67/9	35/2	32/7	NS
ly (0/ 1,2,3)	14/62	9/28	5/34	NS
v (0/ 1,2,3)	31/45	19/18	12/27	NS
ne (0/ 1,2,3)	11/65	7/30	4/35	NS
Size (</⩾; 30 mm)	37/39	23/14	14/25	0.022
IA, IB, IIA/IIB, III	19/57	13/24	6/33	0.0469
Poor/others	13/63	8/29	5/34	NS

ly=lymphatic invasion; M=distant metastasis; N=nodal metastasis; ne=neural invasion; NS=no significant; Poor=poorly differentiated adenocarcinama; T=tumour depth; v=venous invasion.

Patient stage was determined according to UICC TNM classification.
